# Supplementary material for: Pharmacokinetic Analysis of Omomyc Shows Lasting Structural Integrity and Long Terminal Half-Life in Tumor Tissue
Source: Cancers (Basel). 2023 Jan 29;15(3):826. doi: 10.3390/cancers15030826 (PMC9913332; doi:10.3390/cancers15030826)
Supplement: Supplementary file 1 [file cancers-15-00826-s001.zip › cancers-2157469-supplementary.pdf]

Supplementary Materials

A

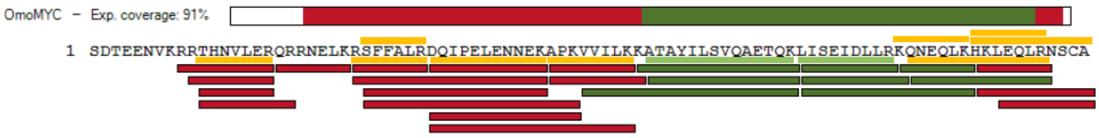

B

1_Omomyc **TEENVKRRTHNVLERQRRNELKRSFFALRDQIPELENNEKAPKVVILKKA**
2_HUMAN_Homo_sapiens TEENVKRRTHNVLERQRRNELKRSFFALRDQIPELENNEKAPKVVILKKA
3_MOUSE_mus_musculus TEENDKRRTHNVLERQRRNELKRSFFALRDQIPELENNEKAPKVVILKKA
**** ***

1_Omomyc **TAYILSVQAETQKLISEIDLLRKQNEQLKHKLEQLRNSCA**
2_HUMAN_Homo_sapiens TAYILSVQAEEQKLISEEDLLRKRREQLKHKLEQLRNSCA
3_MOUSE_mus_musculus TAYILSIQADEHKLTSEKDLLRKRREQLKHKLEQLRNSGA
****** **   ** ** *****   ************* *

C

Sequences (1:2) Aligned. Score: 95.5556
Sequences (1:3) Aligned. Score: 88.8889

Supplementary Figure S1. Sequence coverage of Omomyc and homology with the bHLHLZ of the MYC protein. A) Sequence coverage of Omomyc protein obtained from shotgun LC-MS/MS. The peptide sequences detected are marked in green when unique to Omomyc and in red when shared with MYC. B) Sequence alignment between the 91 amino acid bHLH-LZ domain of Omomyc and the mouse and human MYC proteins. Differences in the amino acid sequence in comparison to Omomyc are indicated by yellow boxes. C) Identity score for the alignment of the bHLH-LZ domain among the different species.

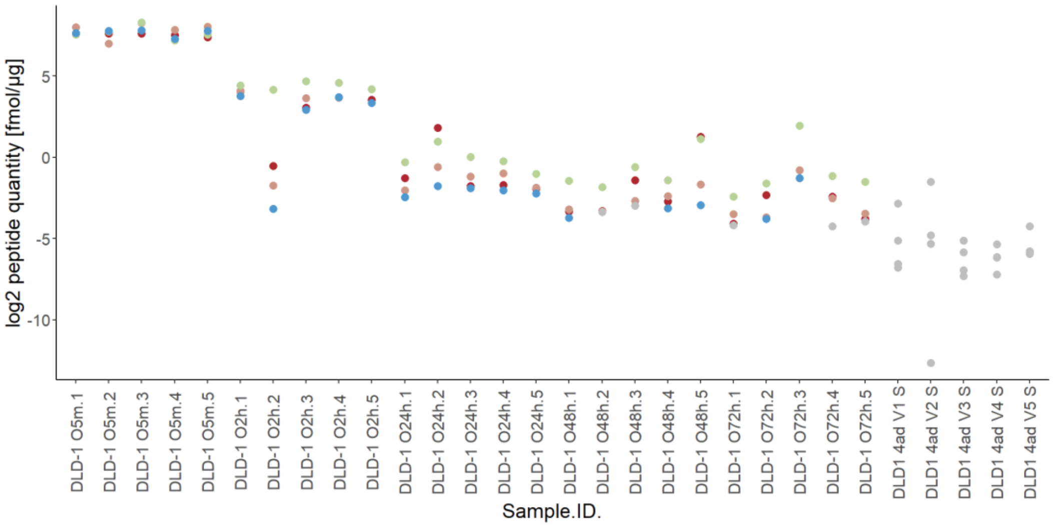

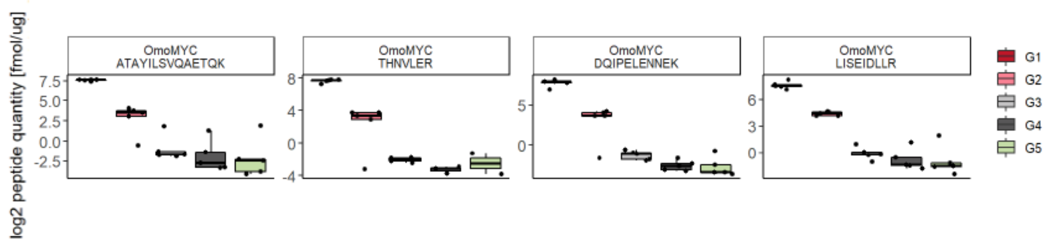

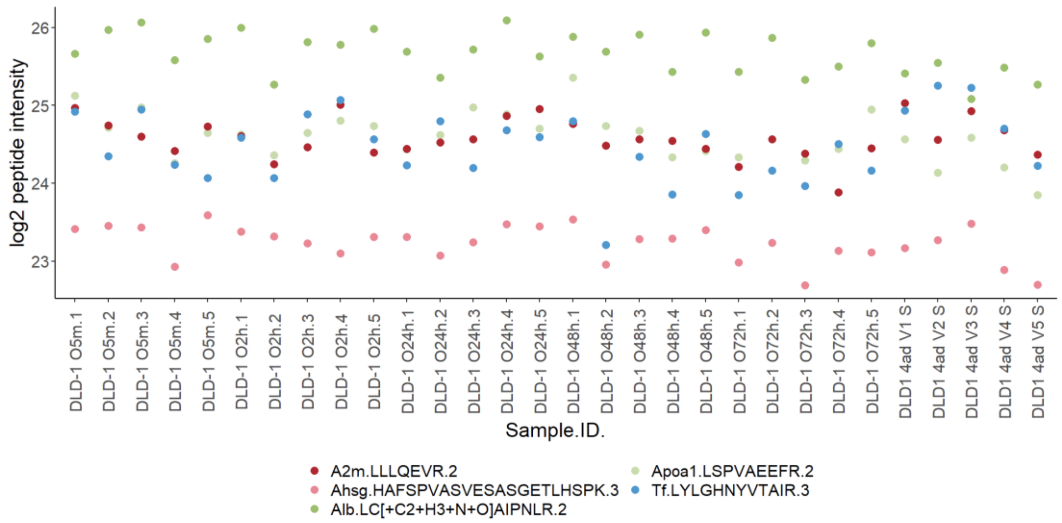

Supplementary Figure S2. Quantification of the reporter peptides in serum samples. a) Quantification of Omomyc reporter peptides. Red: ATAYILSVQAETQK, Green: LISEIDLLR, Pink : DQIPELENNEK, Blue: THNVLER). No detection was observed in 5 vehicle-treated samples (grey dots show not detected signals, q-value > 0.01). b) Box plots showing the absolute quantities of all 4 quantified Omomyc peptides. c) Quantification of 5 control proteins.

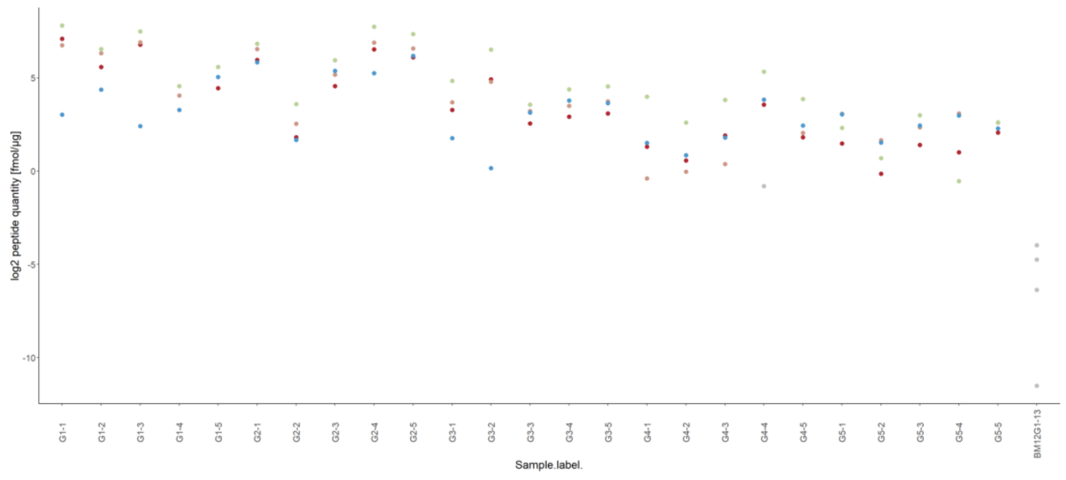

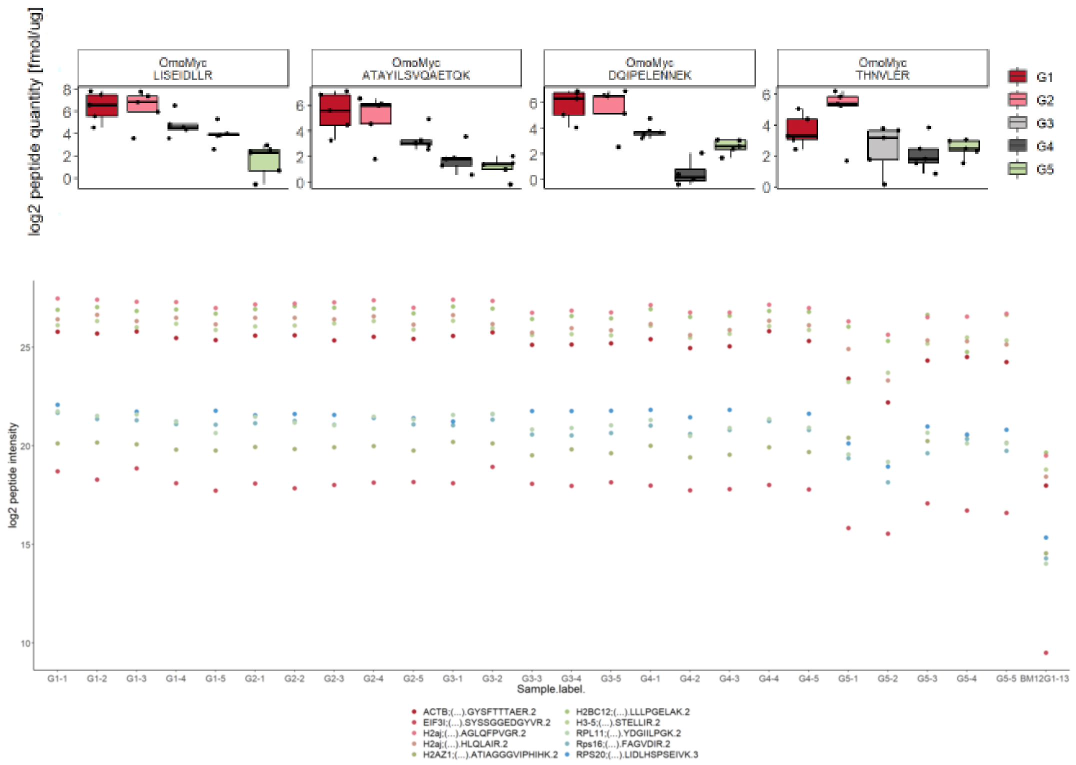

Supplementary Figure S3. Quantification of the reporter peptides in FFPE samples. a) Quantification of Omomyc reporter peptides. Red: ATAYILSVQAETQK, Green: LISEIDLLR, Pink : DQIPELENNEK, Blue: THNVLER). No detection was observed in 5 vehicle-treated samples (grey dots show not detected signals, q-value > 0.01). b) Box plots showing the absolute quantities of all 4 quantified Omomyc peptides. c) Quantification of 10 control peptides (corresponding to 9 proteins).

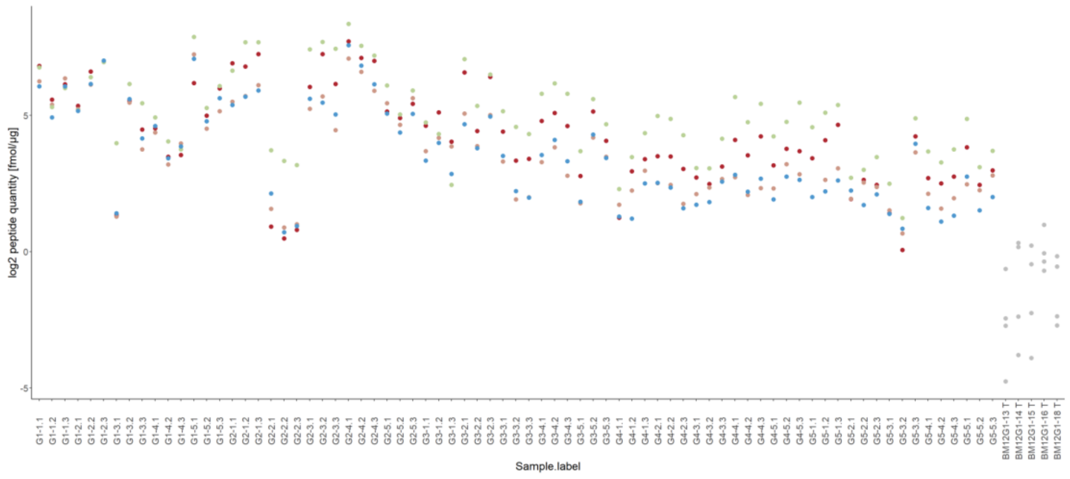

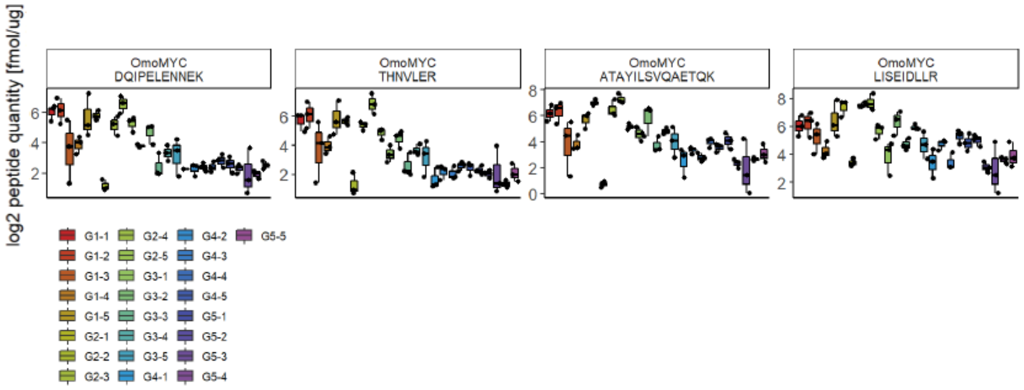

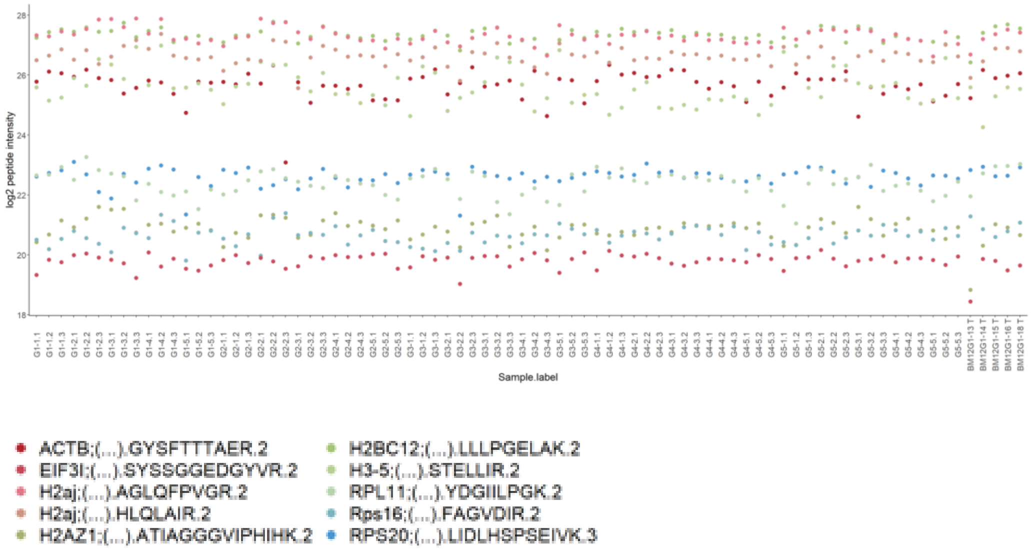

Supplementary Figure S4. Quantification of the reporter peptides in FF samples. a) Quantification of Omomyc reporter peptides. Red: ATAYILSVQAETQK, Green: LISEIDLLR, Pink : DQIPELENNEK, Blue: THNVLER). No detection was observed in 5 vehicle-treated samples (grey dots show not detected signals, q-value > 0.01). b) Box plots showing the absolute quantities of all 4 quantified Omomyc peptides. c) Quantification of 10 control peptides (corresponding to 9 proteins).

Supplementary Table S1. Peptides identified in the shotgun LC-MS/MS of the Omomyc protein. The sequences selected for testing in LC-PRM are identified.

#	Peptide sequence
1	APKVVILK[1]
2	APKVVILKK
3	ATAYILSVQAETQK[1,2]
4	DQIPELENNEK[1]
5	DQIPELENNEKAPK
6	DQIPELENNEKAPKVVILK
7	HKLEQLR[1]
8	HKLEQLRNSCA[1]
9	KATAYILSVQAETQK
10	KQNEQLK[1]
11	LEQLRNSCA
12	LISEIDLLR[1,2]

13	LISEIDLLRK
14	LISEIDLLRKQNEQLK
15	QNEQLKHKLEQLR[1]
16	QRRNELK
17	RRTHNVLER
18	RSFFALR[1]
19	RSFFALRDQIPELENNEK
20	RTHNVLER
21	SFFALR[1]
22	SFFALRDQIPELENNEK
23	SFFALRDQIPELENNEKAPK
24	THNVLER[1]
25	THNVLERQR
26	VVILKKATAYILSVQAETQK

[1] Selected for testing in LC-PRM
[2] SIS peptide available

Supplementary Table S2. List of monitored Omomyc and MYC peptides for absolute quantification and control peptides for label free quantification.

Protein / Gene IDs	Peptide sequence	Quantification	Matrix
Omomyc	ATAYILSVQAETQK	SIS	All
Omomyc	DQIPELENNEK	SIS	All
Omomyc	LISEIDLLR	SIS	All
Omomyc	THNVLER	SIS	All
MYC	SSDTEENVK	SIS	All
H2aj;H2AC6;H2AZ1;H2az1;H2az2;H2AZ2(…)	AGLQFPVGR	Label free	FFPE, FF
H2aj;H2AC6;H2AZ1;H2az1;H2az2;H2AZ2(…)	HLQLAIR	Label free	FFPE, FF
H2AZ1;H2az1;H2az2;H2AZ2	ATIAGGGVIPHIHK	Label free	FFPE, FF
H2BC12;H2BC11;H2bc7;H2bc14;(…)	LLLPGELAK	Label free	FFPE, FF
H3-5;H3C1;H3c1;H3c2;H3-3A;H3-3a;H3-4;H3-2;H3-5;H3C15	STELLIR	Label free	FFPE, FF
ACTB;Actb;Actg1;ACTG1	GYSFTTTAER	Label free	FFPE, FF
EIF3I;Eif3i	SYSSGGEDGYVR	Label free	FFPE, FF
RPL11;Rpl11	YDGIILPGK	Label free	FFPE, FF
Rps16;RPS16	FAGVDIR	Label free	FFPE, FF
RPS20;Rps20	LIDLHSPSEIVK	Label free	FFPE, FF
Alb	LCAIPNLR	Label free	Serum
Ahsg	HAFSPVASVESASGETLHSPK	Label free	Serum
Apoa1	LSPVAEEFR	Label free	Serum
A2m	LLLQEVR	Label free	Serum
Tf	LYLGHNYVTAIR	Label free	Serum
